# Molecular biomarkers in dialysis-related thrombosis and anticoagulation monitoring: thrombin–antithrombin complex, plasmin-α2-antiplasmin complex, soluble thrombomodulin, tPA/PAI-1/tPAIC, and Anti-Xa

**DOI:** 10.3389/fmed.2026.1869337

**Published:** 2026-07-30

**Authors:** Yuning Lin, Huarong Lu, Zhenyi Lv, Songping Chen, Hongwei Jin

**Affiliations:** Medical Laboratory, Xiamen Humanity Hospital, Fujian Medical University, Xiamen, Fujian, China

**Keywords:** Anti-Xa, dialysis, PAI-1, PIC, STM, TAT, tPA, tPAIC

## Abstract

Patients with end-stage renal disease (ESRD) undergoing renal replacement therapies, including hemodialysis, continuous renal replacement therapy (CRRT), and peritoneal dialysis, face a dual risk of thrombosis and bleeding complications. Conventional coagulation monitoring parameters, such as activated partial thromboplastin time (APTT) and prothrombin time (PT), have substantial limitations in uremic patients and fail to accurately reflect their true hemostatic status. In recent years, emerging molecular biomarkers, including thrombin-antithrombin complex (TAT), plasmin-α2-antiplasmin complex (PIC), soluble thrombomodulin (sTM), tissue-type plasminogen activator (tPA), plasminogen activator inhibitor-1 (PAI-1), tPA–PAI-1 complex (tPAIC) and anti-factor Xa activity (Anti-Xa), have been investigated as complementary biomarkers for assessing thrombin generation, fibrinolytic activation, endothelial injury, fibrinolytic dysregulation, and anticoagulant drug effect in selected dialysis-related settings. This review summarizes the biological characteristics of these biomarkers, their clinical utility across different dialysis modalities, recent advances in detection methodologies, and strategies for multimarker monitoring. Current evidence suggests that these biomarkers may provide complementary mechanistic information beyond conventional assays regarding coagulation activation, fibrinolytic status, endothelial injury, and anticoagulant drug effect. However, dialysis-specific outcome evidence remains uneven, and no validated five-marker strategy has yet been established for routine clinical decision-making. Therefore, these biomarkers should currently be viewed as adjunctive and exploratory tools, with potential use in selected scenarios such as CRRT anticoagulation assessment, vascular access risk stratification, and individualized anticoagulant evaluation. However, several challenges remain, including assay standardization, establishment of reference ranges, and cost-effectiveness evaluation. Further prospective studies are needed to validate whether scenario-based single-marker or multimarker interpretation can improve clinically meaningful outcomes, including circuit patency, vascular access survival, bleeding prevention, and individualized anticoagulation management.

## 1 Introduction

### 1.1 Epidemiological characteristics and clinical significance of thrombosis in dialysis patients

With the increasing burden of chronic kidney disease and its progression to kidney failure, kidney replacement therapy has become an essential treatment for patients with advanced kidney failure. According to a Global Burden of Disease Study 2023 systematic analysis, the global number of people with kidney failure with replacement therapy (KFRT) reached 4.59 million in 2023 (1). Among these modalities, maintenance hemodialysis and peritoneal dialysis remain the principal treatment options for patients with chronic kidney failure (1, 2), CRRT is primarily used in critically ill patients with acute kidney injury or hemodynamic instability (3, 4). Although dialysis can partially replace renal function, patients remain in a fragile hemostatic balance throughout treatment, characterized by the coexistence of increased thrombotic risk and a heightened risk of bleeding following anticoagulation. This dilemma directly affects dialysis adequacy, vascular access longevity, hospitalization rates, and survival outcomes (2, 5, 6). Recent systematic evidence indicates that the overall prevalence of thrombosis in dialysis patients is approximately 14.2%, increasing to 16.2% among those with vascular access and 22.8% among patients with arteriovenous grafts (AVGs). These findings suggest that AVGs carry a particularly high thrombotic burden compared with other vascular access types (5). In the setting of CRRT, clotting of the filter or extracorporeal circuit is likewise a major cause of treatment interruption and underdosing. Therefore, achieving a balance between minimizing circuit clotting and avoiding bleeding complications remains a central challenge in dialysis and critical care kidney support (6, 7). At present, the heparin monitoring methods most commonly used in clinical practice include activated partial thromboplastin time (APTT), activated clotting time (ACT), and Anti-Xa activity assays. However, existing reviews have noted that there is still no consensus on the optimal dosing strategy or target range for anticoagulation monitoring in dialysis. In addition, these assays are limited by issues related to accessibility, standardization, interfering factors, and variability in result interpretation, and evidence that they improve clinical outcomes remains insufficient. Therefore, molecular biomarkers that more directly reflect thrombin generation, fibrinolytic activation, endothelial injury, and the pharmacodynamic effects of heparin are increasingly becoming an important focus of research in dialysis anticoagulation monitoring. In recent years, biomarkers such as TAT, PIC, sTM, tPA, PAI-1, tPAIC and anti-factor Xa activity have shown potential value in thrombotic risk stratification and in the assessment of coagulation–fibrinolysis imbalance, thereby providing a new basis for individualized anticoagulation strategies (7–9).

### 1.2 Necessity of molecular biomarkers in thrombosis diagnosis and anticoagulation monitoring

Conventional coagulation tests, such as PT, APTT, FIB, mainly reflect changes in clotting time resulting from coagulation factor deficiency, consumption, or the effects of anticoagulant drugs, and are therefore more suitable for identifying hypocoagulability or overt coagulation disorders. However, their sensitivity and ability to dynamically reflect early hypercoagulability, persistent thrombin generation, and prothrombotic alterations associated with endothelial activation are limited, making them insufficient for early thrombotic risk warning and refined anticoagulation monitoring (10, 11). They therefore have potential value for characterizing prothrombotic states and coagulation–fibrinolysis imbalance, although their dialysis-specific clinical utility requires further validation (12). Molecular biomarkers such as TAT, PIC, sTM, and tPAIC may provide complementary information on thrombin generation, fibrinolytic activation or inhibition, and endothelial injury. Among them, sTM is particularly linked to endothelial dysfunction and has been explored for disease monitoring in DIC, sepsis, stroke, and multiple organ failures (13). However, the combined use of these biomarkers for early thrombotic risk warning and individualized anticoagulation management remains exploratory.

Due to the coexistence of multiple factors such as abnormal platelet function, vascular endothelial damage, chronic inflammatory response, and fibrinolysis imbalance in uremic conditions, traditional coagulation function tests often fail to accurately reflect the true and complex hemostatic status of patients (14, 15). Current studies in ESRD and hemodialysis patients have reported elevated thrombo-inflammatory markers, including tPA, PAI-1, D-dimer, CRP, vWF, and other markers previously linked to thrombin generation and endothelial dysfunction, such as TAT and TM/sTM. These findings support the presence of a chronic thrombo-inflammatory milieu characterized by coagulation activation, endothelial dysfunction, and fibrinolytic dysregulation (16). In anticoagulation monitoring, Anti-Xa activity more specifically reflects the pharmacodynamic effect of unfractionated heparin than APTT-based monitoring and may help optimize dose adjustment and therapeutic response assessment, although its role in dialysis-specific anticoagulation strategies requires further validation (17).

## 2 Pathophysiological basis of dialysis-related thrombosis and anticoagulation monitoring

### 2.1 Characteristics of coagulation abnormalities in dialysis patients

Patients with advanced chronic kidney disease or end-stage renal disease, particularly those undergoing dialysis, exhibit a distinctive hemostatic imbalance characterized by the coexistence of thrombotic tendency and bleeding risk, rather than a simple hypercoagulable or hypocoagulable state (18, 19). Moreover, conventional coagulation assays, such as PT and APTT, may remain within the reference range even when fibrinogen, factor VIII, and von Willebrand factor levels are elevated. This indicates that clotting time–based routine tests are insufficient to fully capture the global and dynamic hemostatic phenotype of patients with chronic kidney disease or end-stage renal disease (18, 19). A prospective observational study showed that plasma levels of vWF:Ag, vWF:RCo, fibrinogen, FVII, FVIII, and D-dimer were elevated in patients with CKD and tended to increase with advancing CKD stage (20); Furthermore, a systematic review and meta-analysis showed that PT and APTT were not significantly different between patients with CKD and healthy controls, whereas thromboelastography detected hypercoagulable changes that were not captured by conventional clotting time–based assays. This supports the limited sensitivity of routine coagulation tests in reflecting the complex hemostatic phenotype of CKD (21). In parallel, chronic inflammation, oxidative stress, and endothelial dysfunction collectively create a pro-inflammatory and prothrombotic vascular milieu in CKD, which may further facilitate activation of procoagulant pathways (19). This process is characterized not only by endothelial injury and vWF upregulation, but also by upregulation of tissue factor, elevated PAI-1 levels, and altered platelet reactivity, which together promote a prethrombotic state marked by concurrent procoagulant activation and impaired fibrinolysis (22). Uremic toxins may directly alter platelet adhesion and aggregation, contributing to abnormal platelet reactivity in patients with CKD or ESRD (23). In addition to their effects on platelet function, tryptophan-derived uremic toxins such as indoxyl sulfate, indole-3-acetic acid, and kynurenine may promote thrombosis by upregulating tissue factor expression. IS and IAA can induce TF expression through AHR-dependent pathways involving NF-κB and MAPK signaling, whereas kynurenine mainly acts through the AHR–TF axis. Increased TF expression may activate the extrinsic coagulation cascade, leading to downstream FXa and thrombin generation and subsequent fibrin formation (24). Furthermore, contact between blood and the artificial surfaces within the extracorporeal circuit can trigger the activation of platelet, complement, and coagulation pathways, thereby further exacerbating thromboinflammation during dialysis (25). This blood incompatibility extends beyond the dialysis membrane to include the tubing, blood-air interface, bubble traps, and pump-induced mechanical damage. Even with heparin anticoagulation, processes such as soluble fibrin formation, complement-dependent leukocyte activation, platelet–neutrophil complex formation, and upregulated tissue factor expression can still take place, resulting in sustained thromboinflammation associated with dialysis (26). At the molecular level, elevated vWF levels together with relatively decreased ADAMTS13 activity provide further evidence of a prothrombotic environment in dialysis patients. Of note, a prospective cohort study of 956 dialysis patients demonstrated that higher vWF levels and lower ADAMTS13 activity were correlated with higher mortality (27). D-dimer levels are frequently elevated in hemodialysis patients even in the absence of clinically apparent thromboembolic events. These elevations may reflect ongoing coagulation–fibrinolysis activity and chronic or recurrent subclinical thrombotic burden during hemodialysis. Therefore, D-dimer results in hemodialysis patients should be interpreted in the clinical context rather than being directly equated with acute thrombotic events (28). Because D-dimer levels are inversely associated with eGFR and tend to be higher in patients with renal impairment, their interpretation should consider renal function and clinical context rather than being regarded as direct evidence of acute VTE in isolation (29). Coagulation abnormalities in CKD and dialysis result from platelet dysfunction, endothelial injury, procoagulant activation, extracorporeal circuit-induced thromboinflammation, and impaired fibrinolysis, collectively contributing to dialysis-related thrombosis and complicating anticoagulation management.

### 2.2 Extracorporeal circulation and the mechanisms of thrombus formation

The extracorporeal circulation used in dialysis is inherently a powerful driver of thromboinflammation. When blood is diverted from its native endothelial environment and exposed to dialysis tubing, membranes, and other synthetic surfaces, various biological responses can be set in motion, involving the activation of coagulation, platelet, complement, and innate immune pathways (25, 30). In CRRT/CKRT, circuit clotting is a major contributor to premature filter loss, therapy interruption, and reduced delivered treatment efficacy, with potential implications for workload and resource use (31). Thrombus formation in extracorporeal circuits is not driven by a single pathway, but rather results from the combined effects of multiple interrelated mechanisms. Direct contact between blood and artificial surfaces can activate the contact pathway, particularly through factor XII-dependent initiation (32). Meanwhile, membrane characteristics, including surface chemistry, hydrophilicity, wettability, and other physicochemical properties, influence the initial adsorption of plasma proteins and thereby modulate subsequent coagulation activation (30, 33). Platelet activation also plays a central role, as platelets can adhere to artificial or protein-coated surfaces through adsorbed plasma proteins such as fibrinogen and von Willebrand factor, thereby promoting platelet activation, aggregation, and thrombus formation (25, 30). Complement activation and its crosstalk with coagulation are likewise key components of dialysis-related biocompatibility responses. During hemodialysis, complement, inflammation, and coagulation do not act independently; instead, they form a self-amplifying loop that sustains thromboinflammation and tissue injury (34, 35). Beyond biomaterial-related mechanisms, hemodynamic factors also contribute to circuit clotting. Low blood flow, local stasis, turbulence, hemoconcentration, and suboptimal vascular access conditions may all shorten circuit patency, while non-pharmacological factors, such as catheter position and flow restriction, may further compromise circuit survival (3, 36).

### 2.3 Differences in thrombotic risk across dialysis modalities

The thrombotic risk and coagulation abnormalities associated with different dialysis modalities should not be simply interpreted as being “absolutely higher” in one modality than another. Rather, they should be understood as reflecting distinct pathophysiological mechanisms specific to each modality (25, 37–39). In patients undergoing hemodialysis (HD), thrombotic risk is primarily associated with vascular access–related complications rather than systemic coagulation abnormalities alone (40, 41). Contact between blood and artificial materials, such as dialysis membranes and tubing, can activate the complement system, platelets, and the coagulation cascade. In addition, the type of vascular access further influences thrombotic risk, with current evidence suggesting that arteriovenous grafts (AVGs) are associated with a higher risk of thrombosis than arteriovenous fistulas (AVFs) (42, 43). In contrast, peritoneal dialysis (PD), including continuous ambulatory peritoneal dialysis (CAPD), does not involve extracorporeal circulation. Reported coagulation-related abnormalities in PD are often reflected by local disturbances of the intraperitoneal coagulation–fibrinolysis balance, particularly increased peritoneal fibrin turnover and altered fibrin generation/degradation during CAPD or peritonitis (44, 45). Several factors, including the uremic milieu (46), the bioincompatibility of conventional glucose-based PD fluids and glucose degradation products (GDPs) (47), long-term peritoneal exposure, and episodes of peritonitis (48), may continuously promote mesothelial injury, inflammatory amplification, vascular alterations, and peritoneal fibrosis, thereby increasing the risk of severe complications such as encapsulating peritoneal sclerosis (EPS). Therefore, HD and PD should be regarded as two dialysis modalities with distinct coagulation and thrombotic profiles, and clinical monitoring and preventive strategies should be individualized according to the specific dialysis modality.

### 2.4 Limitations of conventional anticoagulation monitoring parameters

Patients with chronic kidney disease (CKD), particularly those with ESRD, exhibit a complex coagulation phenotype characterized by the coexistence of hypercoagulability and platelet dysfunction. However, conventional coagulation assays are unable to fully capture these abnormalities (49). Recent studies have shown that standard coagulation test results in dialysis patients do not always correspond with global hemostatic assays or with clinical bleeding and thrombotic events, suggesting that reliance on PT, APTT, and international normalized ratio (INR) alone is insufficient for accurately assessing the true hemostatic status in this population (15, 50, 51). D-dimer also has important interpretive limitations in patients with impaired renal function (52, 53). Although it remains a useful biomarker reflecting cross-linked fibrin formation and fibrinolytic activation, its diagnostic specificity decreases as renal function worsens (29, 53). Studies have shown that D-dimer levels increase with declining renal function, and its specificity for excluding pulmonary embolism decreases significantly as renal impairment progresses (54). Therefore, in dialysis patients, conventional coagulation and anticoagulation monitoring parameters should be regarded primarily as tools for basic screening or supportive assessment, rather than as the sole basis for evaluating thrombotic risk or anticoagulation intensity. Combining these parameters with Anti-Xa activity, TAT, PIC/PAP, TM/sTM, tPA-PAI-1 complex, or global hemostatic assays may provide complementary mechanistic information for individualized risk assessment (55–57).

### 2.5 Global hemostatic assays and rationale for selecting molecular biomarkers

Global hemostatic assays, including viscoelastic tests such as thromboelastography and rotational thromboelastometry, as well as thrombin generation assays, provide complementary information beyond clotting time. Thromboelastography (TEG), rotational thromboelastometry (ROTEM) characterize clot initiation, propagation, clot strength, and fibrinolysis, whereas thrombin generation assay (TGA) quantifies the kinetics and overall amount of thrombin generated (58–61). Compared with conventional clotting-time assays such as PT and APTT, global hemostatic assays may provide a more comprehensive representation of the net hemostatic phenotype in patients with CKD or dialysis-related hemostatic abnormalities by capturing abnormalities in thrombin generation, clot kinetics, clot strength, and fibrinolysis that may not be apparent from routine clotting times (19, 21, 49, 51). Nevertheless, these assays do not replace molecular biomarkers or drug-specific activity assays, because each approach addresses a different aspect of hemostatic assessment. Global assays characterize the overall *ex vivo* hemostatic phenotype, whereas molecular biomarkers provide pathway-specific information on coagulation activation, fibrinolysis, endothelial injury, or anticoagulant drug effect.

However, global hemostatic assays, molecular biomarkers, and drug-specific activity assays answer different clinical questions (62). TAT reflects thrombin generation and coagulation activation, PIC/PAP reflects plasmin generation and fibrinolytic activation, sTM reflects endothelial perturbation, and tPA-, PAI-1-, or tPAIC-related measurements reflect fibrinolytic regulation (63, 64). Anti-Xa activity primarily reflects the pharmacodynamic activity of an anticoagulant when measured with an appropriate drug-specific calibration (65, 66). Thus, TGA (67) assesses thrombin-generating potential, whereas TAT represents a circulating footprint of recent *in vivo* coagulation activation (61, 68). PIC/PAP reflects plasmin generation and fibrinolytic activation (69), sTM reflects endothelial injury and impairment of the endothelial anticoagulant barrier (13), tPA/PAI-1/tPAIC reflects fibrinolytic regulation and PAI-1-mediated tPA inhibition (70), and Anti-Xa activity reflects the pharmacodynamic effect of unfractionated heparin (UFH), low-molecular-weight heparin (LMWH), or factor Xa inhibitors (71). Thus, global assays may be useful for assessing the overall coagulation phenotype, whereas molecular biomarkers may help identify which component of the coagulation–fibrinolysis–endothelial axis is altered.

## 3 Biological basis and analytical characteristics of the five biomarkers

These biomarkers were selected because they represent five complementary domains directly relevant to the scope of this review: thrombin generation, fibrinolytic activation, endothelial injury, fibrinolytic regulation, and anticoagulant drug effect. Other biomarkers, including vWF, ADAMTS13, complement activation products, P-selectin, and additional platelet activation markers, are also relevant to dialysis-related thromboinflammation (27, 72, 73). However, they were not selected as core biomarkers because they primarily index broader endothelial-platelet, complement, or cellular platelet-activation pathways beyond the focused coagulation-fibrinolysis-anticoagulant framework adopted in this review (74, 75). Molecular biomarkers relevant to dialysis-related thrombosis and anticoagulation monitoring can be understood from five complementary dimensions: thrombin generation, plasmin formation, endothelial injury, fibrinolytic inhibition, and heparin pharmacodynamics. Compared with conventional clotting-time assays, these biomarkers provide a more direct reflection of underlying pathophysiological processes. However, their interpretation should be contextualized according to dialysis modality, sampling time, analytical platform, and the specific clinical scenario. The biological significance, detection methods, major dialysis-related applications, and key limitations of these five biomarkers are summarized in Table 1.

**Table 1:** Key molecular biomarkers in dialysis-related thrombosis and anticoagulation monitoring.

Biomarker	Main biological meaning	Detection methods	Main use in dialysis	Key limitations
TAT	Thrombin generation/coagulation activation	ELISA; CLIA/CLEIA	Assess coagulation activation, circuit clotting tendency, and vascular access risk	Sensitive to sampling; platform-dependent reference intervals
PIC/PAP	Plasmin generation/fibrinolytic activation	ELISA; CLIA/CLEIA	Evaluate fibrinolytic response and coagulation–fibrinolysis balance; useful with TAT or TAT/PIC ratio	Not disease-specific; cut-offs in dialysis remain unclear
sTM	Endothelial injury/impaired endothelial anticoagulant barrier	EIA/ELISA; CLIA/CLEIA	Reflect endothelial dysfunction, vascular injury, and possible access-related risk	Affected by renal function, inflammation, and assay platform
PAI-1/tPA/tPAIC	PAI-1-mediated tPA inactivation/fibrinolytic inhibition	ELISA; CLIA/CLEIA	Indicate impaired fibrinolytic regulation and low-fibrinolytic tendency	Should be interpreted with PIC, D-dimer and sTM
Anti-Xa activity	Anticoagulant effect against factor Xa	Chromogenic Anti-Xa assay	Monitor UFH, LMWH, or Xa inhibitor exposure; guide individualized anticoagulation	Requires drug-specific calibration; does not directly reflect thrombosis

### 3.1 Thrombin-antithrombin complex

#### 3.1.1 Biological characteristics and detection methods of thrombin–antithrombin complex

TAT is formed through the equimolar binding of thrombin to antithrombin and reflects *in vivo* thrombin generation. It is therefore regarded as a sensitive molecular biomarker of coagulation activation. Compared with conventional coagulation parameters, such as PT and APTT, TAT is more closely linked to thrombin formation and may indicate coagulation activation before overt abnormalities appear in routine clotting-time assays. Recent methodological and clinical studies have supported the analytical feasibility and clinical relevance of TAT measurement for detecting early coagulation activation and thrombotic risk (76–78). Because free thrombin has an extremely short circulating half-life and is difficult to measure reliably in clinical practice, TAT provides a more stable and detectable surrogate for assessing *in vivo* thrombin generation. TAT can be detected shortly after coagulation is initiated, and its relatively short circulating half-life, reported at approximately 3–15 min, supports its use as a dynamic marker of recent or ongoing coagulation activation (78, 79). A recent performance-verification study of a high-sensitivity automated chemiluminescence platform demonstrated acceptable precision, trueness, carryover, linearity, and reference-interval verification for TAT measurement, supporting its suitability for clinical sample testing (80). However, reference intervals may vary across analytical platforms, populations, and laboratories. Therefore, a single “normal value” should not be applied mechanically; instead, TAT results should be interpreted according to the specific assay system used and the local laboratory reference range.

#### 3.1.2 Clinical significance and dialysis-related applications

In dialysis patients, the primary value of TAT lies in its ability to reflect thrombin generation induced by extracorporeal circulation. During hemodialysis, contact between blood and artificial interfaces, such as dialysis membranes, catheters, tubing surfaces, and air–blood interfaces, can activate the complement system, platelets, and the coagulation cascade, thereby sustaining a low-grade thromboinflammatory response. Membrane biocompatibility and blood–biomaterial interactions have been identified as important mechanisms of coagulation activation during dialysis, and TAT generation has been used as a hemocompatibility endpoint (26, 81, 82). In addition, TAT can be used to monitor dynamic coagulation changes during a single dialysis session and to evaluate the effects of anticoagulation strategies. A randomized phase II study in patients with end-stage kidney disease found that, in the placebo group undergoing hemodialysis, TAT increased from 6.1 ng/mL to 71.8 ng/mL, indicating that even a single hemodialysis treatment can markedly induce systemic thrombin generation (83). TAT may also have prognostic relevance for vascular access outcomes. A prospective study involving 462 hemodialysis patients showed that an elevated TAT/PIC ratio was an independent risk factor for vascular access failure and could predict clinical outcomes such as revascularization or access repair in hemodialysis patients (84). Furthermore, a randomized crossover study reported that baseline TAT levels were higher in dialysis patients than in healthy controls, and that TAT increased further during dialysis despite systemic anticoagulation. These findings suggest that TAT may serve as one useful marker for comparing the degree of coagulation activation associated with different modern dialysis membranes (82). Table 2 summarizes representative studies evaluating TAT in dialysis populations. These studies highlight the value of TAT as a marker of thrombin generation and coagulation activation during hemodialysis, particularly in relation to extracorporeal circulation, vascular access dysfunction, dialyzer biocompatibility, and anticoagulation strategies.

**Table 2:** Sampling and assay characteristics of TAT monitoring in dialysis-related studies.

Author	Study population/groups	Sampling time	Blood sampling site	Assay method	Main findings	References
Stegmayr et al., 2024	20 long-term HD patients; 60 dialysis sessions	0, 30, and 180 min	Arterial limb/pre-dialyzer line	Commercial kit	TAT exceeded the reference limit in 58% of predialysis samples and correlated dynamically with D-dimer, indicating chronic coagulation activation.	(28)
Francois et al., 2020	10 maintenance HD patients in a randomized crossover study of PP, PMMA, and AN69ST membranes; 20 healthy controls	Before HD/UFH and at 5, 15, 30, 90, and 240 min	AVF samples in HD patients; venipuncture in controls	Commercial ELISA	Baseline TAT was higher in HD than controls; TAT increased from 90 min and was significant at 240 min. PMMA showed a relatively lower increase.	(82)
Verbout et al., 2024	36 ESRD patients on maintenance HD randomized to placebo, AB002 1.5 ug/kg, or AB002 3 ug/kg (*n* = 12 each)	1 h before HD and at the end of HD on dosing and non-dosing days	NR	ELISA	Heparin-free HD markedly increased TAT; AB002 3 ug/kg substantially reduced dialysis-induced TAT generation.	(83)
Hasuike et al., 2019	462 maintenance HD patients undergoing vascular access intervention; access failure (*n* = 162) vs. patent access (*n* = 300)	Before intervention	NR	Enzyme immunoassay	Access failure was associated with higher TAT and TAT/PIC ratio; TAT/PIC ≥ 4.17 × 10^−3 independently predicted access failure.	(84)
Sagedal et al., 2001	12 chronic HD patients (6 on warfarin, 6 without warfarin) vs. 10 predialysis CRF patients; 84 HD sessions	Before HD, 3 h, and 4 h	Dialysis arterial line; venous stasis avoided	ELISA	TAT increased during HD. Warfarin attenuated TAT and F1 + 2 generation; TAT correlated with circuit/air-trap clotting.	(178)
Malyszko et al., 2001	23 CAPD patients and 24 HD patients vs. 13 healthy controls	HD: before dialysis/heparin; CAPD: fasting outpatient sample; controls: morning sample	Venous blood	Commercial assays	TAT and F1 + 2 were higher in dialysis patients than controls and higher in CAPD than HD; TAT correlated with TF and vWF.	(179)
Milburn et al., 2013	70 HD patients vs. 78 healthy controls	Immediately before HD and before heparin; controls at rest	Antecubital vein	ELISA	TAT was significantly higher in HD patients and was inversely associated with AVF patency.	(180)
Jiao et al., 2021	122 maintenance HD patients with AVF stenosis; restenosis vs. patent access after PTA	Before PTA	Venous blood	ELISA	Restenosis patients had higher TAT. TAT predicted restenosis after PTA (AUC 0.792; cutoff 4.270 ug/L).	(181)
Eloot et al., 2025	9 chronic HD patients in a randomized crossover comparison of four dialyzers	0, 10, 30, 60, 180, and 240 min	Dialyzer inlet and outlet blood lines	ELISA	TAT increased over dialysis time; no significant difference in TAT increase among the four dialyzers.	(182)
Orsag et al., 2021	30 chronic HD patients randomized to standard citrate (*n* = 15) or increased citrate dose (*n* = 15)	t0, 30-min venous line, and 4-h arterial/venous lines	Vascular access and arterial/venous dialysis lines	Enzyme immunoassay	TAT increased after 4 h in both groups; increasing citrate dose did not further reduce TAT generation.	(183)
ElSayed et al., 2023	20 maintenance HD patients in a crossover comparison of Helixone FX80 vs. Platinum H4; 10 healthy controls	Before HD and at 4 h/end of HD	Fistula needle	Commercial ELISA	TAT rose significantly after HD; absolute and percentage increases were similar between the two dialyzers.	(184)

### 3.2 Plasmin-α2-antiplasmin complex

#### 3.2.1 Biological characteristics and detection methods of plasmin-α2-antiplasmin complex

PIC, also known as plasmin–antiplasmin complex (PAP), is a stable complex formed by the binding of plasmin to its principal physiological inhibitor, α2-antiplasmin. Because PIC/PAP directly reflects *in vivo* plasmin generation, it is regarded as a relatively specific molecular biomarker of fibrinolytic system activation (85, 86). α2-Antiplasmin is a key serine protease inhibitor involved in the regulation of fibrinolysis and is the main endogenous inhibitor of plasmin *in vivo*. Once plasmin is generated, it is rapidly inhibited by α2-antiplasmin, forming a stable 1:1 plasmin-α2-antiplasmin complex. Therefore, PIC/PAP can serve as a molecular marker reflecting recent or dynamic activation of plasmin generation and fibrinolysis *in vivo* (87, 88). PIC/PAP differs biologically from D-dimer. D-dimer is a downstream degradation product generated after plasmin-mediated breakdown of cross-linked fibrin, and therefore reflects the combined process of fibrin formation and subsequent fibrinolytic degradation (89, 90). By contrast, PIC/PAP is rapidly formed through the binding of plasmin to α2-antiplasmin and thus more directly reflects the upstream event of plasmin generation. Accordingly, in clinical scenarios requiring dynamic identification of recent or early fibrinolytic activation, PIC/PAP may indicate fibrinolytic activity more sensitively than D-dimer (89, 91).

PIC/PAP is mainly detected by ELISA or automated chemiluminescence-based immunoassays such as CLEIA/CLIA (57, 92). Its reference interval is assay- and population-dependent; in healthy older adults, PIC increases with age, supporting age-specific interpretation (76). Therefore, results should be interpreted using locally validated reference ranges. Clinically, PIC/PAP reflects fibrinolytic activation and may detect early fibrinolytic changes more directly than D-dimer or FDPs in selected settings. In acute cerebral infarction, higher PIC levels were observed in patients with massive cerebral infarction than in those with non-massive infarction, and PIC showed diagnostic value for early identification of massive cerebral infarction (86).

#### 3.2.2 Clinical significance and dialysis-related applications

In dialysis patients, the main clinical significance of plasmin-α2-antiplasmin complex (PIC) lies in its ability to reflect an imbalance between coagulation and fibrinolysis, thereby helping to assess thrombosis-related risks, particularly the risk of vascular access events. PIC may also be relevant to vascular access outcomes. One study showed that patients who developed vascular access failure had a higher TAT/PIC ratio, and that an elevated TAT/PIC ratio was an independent risk factor for vascular access failure, with a hazard ratio of 1.58 and a *p* value of 0.03 (84). Previous dialysis studies have shown abnormalities in coagulation and fibrinolysis markers, including TAT, PIC/PAP, PAI-1, and D-dimer. In patients undergoing HD or CAPD, higher PIC/PAP and D-dimer levels were observed in those with ischemic heart disease, suggesting a potential association between fibrinolytic activation and cardiovascular complications (93). Overall, PIC/PAP reflects plasmin generation and fibrinolytic activation rather than thrombus formation itself. In dialysis patients, it may serve as an adjunctive marker of coagulation–fibrinolysis imbalance, especially when combined with coagulation markers such as TAT. However, its interpretation may be affected by circulating half-life, assay platform, sampling strategy, and the lack of validated thresholds. Therefore, current evidence supports PIC/PAP as a mechanistic marker for risk assessment, but not as a stand-alone indicator for anticoagulant dose adjustment; further prospective studies are needed to clarify its clinical utility (84, 94, 95). A recent methodological study reported a rapid chemiluminescence immunoassay using novel monoclonal antibodies for detecting plasmin–α2-antiplasmin complex (96). In maintenance hemodialysis patients, one study found that the TAT/PIC ratio and FDPs were independent risk factors for vascular access thrombosis (97), while another study also identified the TAT/PIC ratio as an independent predictor of vascular access thrombosis (98). In pediatric hemodialysis patients, PAP and D-dimer levels were higher than those in healthy controls, suggesting activation of fibrinolysis in children with ESRD. However, the study mainly supported D-dimer as a marker related to vascular access patency, whereas the predictive value of PAP was limited after adjustment (99). PIC/PAP has been evaluated in several dialysis studies as a marker of fibrinolytic activation and vascular access outcomes. Although PIC/PAP is often elevated in dialysis patients, its predictive value for access thrombosis appears limited when used alone; combined indices such as the TAT/PIC ratio may provide better risk stratification. Representative studies are summarized in Table 3.

**Table 3:** Representative studies evaluating PIC/PAP in dialysis populations.

Author	Study population	Sampling time point	Sampling site	Assay method	Main findings	References
Hasuike et al., 2019	HD patients after access intervention: access failure (*n* = 162) vs. patent access (*n* = 300).	Before vascular access intervention.	NR.	Latex photometric immunoassay.	PIC did not differ significantly between groups; a higher TAT/PIC ratio was associated with access failure.	(84)
Sagedal et al., 2001	Chronic HD (*n* = 12; 84 sessions; 6 warfarin, 6 no warfarin) and predialysis CRF controls (*n* = 10).	Before HD and at 3 and 4 h; controls had a single sample.	HD arterial line; sampling without venous compression.	ELISA.	PAP increased during HD, supporting dialysis-related activation of fibrinolysis.	(178)
Malyszko et al., 2001	CAPD (*n* = 23), HD (*n* = 24), and healthy controls (*n* = 13).	HD: before dialysis and before heparin; CAPD/control: morning fasting samples.	Venous blood.	Commercial Technoclone kit.	PAP was higher in dialysis patients than controls, indicating fibrinolytic activation; CAPD showed lower PAP than HD.	(179)
Ling et al., 2022	Access occlusion (*n* = 23) vs. patent access (*n* = 54).	Patent group: at follow-up end; occlusion group: after occlusion and before treatment.	Antecubital vein.	Latex photometric immunoassay.	PIC was lower in the occlusion group; an elevated TAT/PIC ratio was an independent risk factor for access thrombosis.	(97)
Wang et al., 2020	Access thrombosis (*n* = 65) vs. patent access (*n* = 123).	Before vascular access intervention.	NR.	Latex photometric immunoassay.	An elevated TAT/PIC ratio was associated with access thrombosis and showed good predictive performance (AUC 0.894).	(98)
Fadel et al., 2016	Children on maintenance HD (*n* = 55) and healthy controls (*n* = 20); access-failure and AVF-thrombosis subgroups.	Immediately before HD and before heparin.	Antecubital vein.	ELISA.	PAP was higher in HD children than controls (883.17 +/− 630.21 vs. 163.89 +/− 43.23 ng/mL) and correlated with D-dimer, but was not an independent predictor after adjustment.	(99)
Kushiya et al., 2003	Maintenance HD patients (*n* = 84) and healthy controls (*n* = 20).	Immediately before HD.	NR.	Commercial PIC test.	Predialysis PIC was higher in HD than controls (1.00 +/− 0.52 vs. 0.49 +/− 0.21 ug/mL), with a mild upward trend by dialysis vintage.	(138)

### 3.3 Soluble thrombomodulin

#### 3.3.1 Biological characteristics and detection methods of sTM

Thrombomodulin is a type I transmembrane glycoprotein predominantly expressed on the surface of vascular endothelial cells and serves as an important receptor for thrombin. Upon binding to sTM, thrombin undergoes a functional shift in substrate specificity, promoting the activation of protein C and contributing to the regulation of fibrinolysis, complement activation, and inflammation. Through these mechanisms, sTM plays a key role in maintaining vascular homeostasis and endothelial quiescence (100). In addition to its membrane-bound form, TM can be released from the endothelial cell surface into the circulation as soluble thrombomodulin (sTM), which lacks the transmembrane domain. This release may occur through proteolytic cleavage or chemical, oxidative, inflammatory, and physical stress-related endothelial injury. Therefore, elevated circulating sTM levels are generally regarded as a marker of endothelial injury or endothelial dysfunction (13). At present, circulating sTM is routinely quantified using immunological assays, with sandwich enzyme-linked immunosorbent assay (ELISA) serving as the most widely used method for measuring sTM antigen concentrations (101, 102). However, different assay reagents may recognize different epitopes or fragments of sTM. Therefore, sTM results should be interpreted cautiously according to the analytical platform used and the relevant clinical context.

#### 3.3.2 Clinical significance and dialysis-related applications

Soluble thrombomodulin is the circulating form of thrombomodulin shed from the endothelial cell surface. It is currently regarded more as a surrogate biomarker of endothelial injury and impairment of the endothelial anticoagulant barrier than as a specific marker of thrombosis itself (13). Studies have shown that, in sepsis-induced coagulopathy (SIC) and disseminated intravascular coagulation (DIC), endothelial injury promotes the shedding of cell-surface thrombomodulin, while elevated plasma sTM levels reflect disruption of anticoagulant pathways and endothelial dysfunction (103, 104). Higher sTM levels have been associated with septic shock, sepsis-induced DIC, and poor clinical outcomes (105, 106). Similarly, in acute respiratory distress syndrome (ARDS), elevated sTM has been linked to increased in-hospital mortality, suggesting its potential value as a candidate biomarker of endothelial injury and disease severity (107). In kidney disease, one clinical study showed that sTM levels increased progressively with declining renal function and were positively correlated with serum creatinine, suggesting that sTM may be associated with endothelial injury and renal impairment in this population (108). Early studies in patients undergoing regular dialysis found that basal plasma immunoreactive thrombomodulin levels were significantly higher than those in healthy controls (109). Borawski et al. reported that, in 100 chronic hemodialysis patients, sTM levels were significantly higher than those in healthy controls. They noted that sTM is a conventional marker of endothelial injury, but its levels in dialysis patients may be influenced by multiple factors; therefore, sTM may indicate endothelial injury in this population, but should not be simply interpreted as a marker of a single thrombotic event (110). Borawski et al. further showed that TM levels in hemodialysis patients were associated with vWF, liver function markers, and erythropoietin use, suggesting that circulating TM may reflect overlapping endothelial, hepatic, and hemostatic abnormalities (111). sTM may also be relevant to vascular access dysfunction. A retrospective study found that serum sTM, platelet-activating factor (PAF), and CD62P levels were associated with arteriovenous fistula (AVF) function, and that all three markers were risk factors for vascular access failure. Their combined model predicted vascular access failure with an AUC of 0.879, suggesting that sTM may participate in, or reflect, processes related to endothelial injury, inflammation, and thrombosis within vascular access (112). Tadokoro et al. reported that sTM was independently associated with T50, suggesting a possible link between sTM and vascular calcification-related processes in hemodialysis patients (113). Overall, sTM is commonly elevated in dialysis patients and may reflect endothelial injury, vascular dysfunction, and related hemostatic disturbances. Its dialysis-related clinical associations, including vascular access dysfunction and calcification-related risk, are summarized in Table 4.

**Table 4:** Summary of thrombomodulin studies in dialysis populations.

Author	Study population	Sampling time point	Blood sampling site	Assay method	Main findings	References
Kushiya et al., 2003	84 maintenance HD patients; 20 healthy controls.	Immediately before HD.	NR	NR	TM was significantly higher in HD patients than in controls, with no clear association across HD duration, Fontaine score, ABI/API, or ACI strata.	(138)
Borawski et al., 2001	100 stable, non-diabetic maintenance HD patients; 30 age- and sex-matched healthy controls.	Fasting predialysis sampling on a midweek dialysis morning.	Arterial outlet of the AVF.	ELISA.	sTM was about fivefold higher in HD patients. Positive hepatitis markers, UFH use, EPO therapy, and higher predialysis SBP independently predicted elevated sTM.	(110)
Jiang et al., 2024	160 maintenance HD patients with AVF and 150 healthy controls; HD patients were stratified by AVF status.	NR	Venous blood.	ELISA.	TM was higher in AVF patients and increased with access dysfunction; TM predicted AVF failure (AUC 0.751; OR 5.034).	(112)
Tadokoro et al., 2024	49 adult maintenance HD patients.	At the start of each dialysis session.	Arterial site of the AVF.	Chemiluminescent enzyme immunoassay	Median sTM was 67.1 U/mL; sTM was positively associated with T50 and remained an independent correlate of T50.	(113)
Kushiya et al., 2003	133 chronic renal failure patients, 75 hyperlipidemia patients, and 73 healthy controls.	Immediately before HD.	NR	NR	In HD patients, TM levels did not differ significantly by Ox-LDL or M-LDL strata.	(185)
Jacobson et al., 2002	CRF patients (*n* = 25), HD patients (*n* = 10), PD patients (*n* = 20), and healthy controls (*n* = 27).	Before dialysis in HD patients; during outpatient assessment in CRF and PD patients.	NR	Commercial ELISA.	sTM was significantly higher in all renal disease groups than in controls; sTM correlated positively with sVCAM-1 and negatively with leukocyte CD11b expression.	(186)

### 3.4 tPA/PAI-1/tPAIC

#### 3.4.1 Biological characteristics and detection methods of tPA/PAI-1/tPAIC

tPA is a key serine protease that initiates the fibrinolytic system. It is mainly synthesized and released by vascular endothelial cells and catalyzes the conversion of plasminogen to plasmin, thereby promoting fibrin degradation (114, 115). This process is enhanced when tPA and plasminogen assemble on fibrin or cellular surfaces, allowing fibrinolysis to occur mainly at sites of fibrin formation (116–119). PAI-1, a member of the serine protease inhibitor superfamily, is the principal physiological inhibitor of both tPA and urokinase-type plasminogen activator (u-PA) (120). PAI-1 forms enzyme–inhibitor complexes with tPA or u-PA, thereby limiting the conversion of plasminogen to plasmin, suppressing fibrin degradation, and playing a central regulatory role in maintaining the balance between coagulation and fibrinolysis (64, 70). tPA antigen is usually detected by immunoassays such as ELISA, whereas tPA activity can be assessed by chromogenic, amidolytic, or clot-lysis-based functional assays (118, 119). PAI-1 can be measured by antigen-based and activity-based assays. PAI-1 antigen is commonly detected using immunoassays, such as ELISA or automated immunoassay platforms, whereas active PAI-1 can be measured by functional assays that evaluate its inhibitory effect on plasminogen activators (64, 119, 121). tPAIC is mainly measured by immunoassays such as ELISA or automated immunoassay platforms. It reflects the formation of a stable complex between tPA and PAI-1, indicating that part of circulating tPA has been inhibited. Because tPAIC is affected by both tPA release and PAI-1 availability, it should be interpreted together with tPA and PAI-1 antigen/activity levels (70, 119, 122).

#### 3.4.2 Clinical significance and dialysis-related applications

Fibrinolytic abnormalities in dialysis patients mainly reflect an imbalance between endothelial tPA release and PAI-1-mediated inhibition. Long-term HD and PD may show a hypofibrinolytic background due to reduced tPA activity or increased PAI-1, whereas a single HD session may acutely increase tPA through extracorporeal circuit stimulation. Thus, tPA should be interpreted according to dialysis modality, sampling time, and PAI-1 status (123). In chronic HD, predialysis D-dimer is often elevated and correlates with tPA activity, TAT, interdialytic weight gain, and ultrafiltration rate, indicating a fibrinolytic response to chronic thrombotic burden (28). Allen et al. reported increased PAI-1 antigen and functional PAI-1 in ESRD patients on HD, with PAI-1 antigen correlated with tPA and endogenous glycosaminoglycans, suggesting endothelial injury-related fibrinolytic dysregulation (16). Karumanchi et al. found that PAI-1 levels were higher in ESRD patients than in healthy controls but did not differ between patients with and without cardiorenal syndrome (CRS), suggesting that PAI-1 is associated with ESRD itself rather than CRS severity (124). Costa et al. reported altered PAI-1, tPA, and D-dimer profiles in HD patients, with catheter users showing higher inflammatory activation and higher D-dimer and tPA levels than AVF users, suggesting a greater thrombogenic risk associated with CVC access (125). Trimarchi et al. further found markedly elevated PAI-1 activity in HD patients, although PAI-1 alone did not clearly predict access thrombosis (126). The tPA/PAI-1 axis may also change acutely during HD. Earlier studies showed that tPA activity may increase after a single dialysis session, while other fibrinolytic markers changed less obviously (127). Marney et al. demonstrated that HD increased tPA and PAI-1, and that the postdialysis rise in PAI-1 was partly mediated by bradykinin B2 receptor signaling (128). Sosinska-Zawierucha et al. found that post-HD serum increased tPA secretion and the tPA/PAI-1 ratio in venous endothelial cells, suggesting dialysis-related endothelial fibrinolytic activation (129). Sosinska-Zawierucha et al. found that serum collected after HD increased tPA secretion and the tPA/PAI-1 ratio in cultured venous endothelial cells, suggesting dialysis-related activation of endothelial fibrinolytic potential, particularly in venous endothelial cells (130). *In vitro*, serum and effluent dialysate from CAPD patients increased PAI-1 release and reduced the tPA/PAI-1 ratio in human coronary artery endothelial cells, without significantly altering tPA secretion (131). In peritoneal mesothelial cells, iron isomaltoside reduced tPA synthesis and increased PAI-1 production, decreasing the tPA/PAI-1 ratio; this effect was prevented by N-acetylcysteine (132). PAI-1 may also have prognostic and structural relevance in PD. Arikan et al. found that plasma PAI-1 > 41 ng/mL independently predicted cardiovascular mortality and cardiovascular events in prevalent PD patients (133). Lopes Barreto et al. reported higher effluent PAI-1 appearance rates before EPS diagnosis, supporting its value as a marker of progressive peritoneal fibrosis (134). Hao et al. similarly found that effluent PAI-1 increased during PD and was associated with peritoneal small-solute transfer rate (135). However, Cura et al. found no significant association between PAI-1 gene variants and suspected peritoneal fibrosis, suggesting limited value of PAI-1 genotyping (136). In acute blood purification, Wang et al. showed that PAI-1 decreased and D-dimer increased during CBP, with a greater PAI-1 reduction under heparin anticoagulation, suggesting dynamic coagulation–fibrinolysis changes during acute extracorporeal therapy (137). The tPAIC reflects PAI-1-mediated tPA inactivation and restricted fibrinolysis initiation (70). Kushiya et al. found that although overall tPAIC levels were lower in HD patients than in controls, tPAIC was higher in patients with severe peripheral arterial disease or greater aortic calcification, suggesting a relationship with endothelial injury and atherosclerosis-related hemostatic abnormalities (138). Overall, tPA, PAI-1, and tPAIC should be interpreted in relation to dialysis modality, vascular access, sampling time, anticoagulation strategy, and other coagulation-fibrinolysis markers. tPA-, PAI-1-, and tPAIC-related markers reflect fibrinolytic imbalance and vascular risk in dialysis and blood purification settings. Representative studies are summarized in Table 5.

**Table 5:** Summary of tPA/PAI-1/tPAIC related findings in dialysis and blood purification studies.

Author	Study population/comparison	Sampling time	Sample/source	Assay	Key finding/manuscript relevance	References
Allen et al., 2023	ESRD HD patients (*n* = 73) vs. healthy nonsmoking controls (*n* = 10); cardiovascular subgroup analyses.	Single cross-sectional blood sampling; relation to dialysis timing NR.	Citrated whole blood; site NR.	ELISA-based assays	PAI-1 antigen, functional PAI-1 and tPA were all higher in ESRD. PAI-1 correlated with tPA, CRP and GAGs and inversely with D-dimer.	(16)
Stegmayr and Lundberg, 2024	Twenty chronic HD patients; 60 HD sessions; comparison of three low-flux dialysis modes.	Predialysis, 30 min and 180 min after HD start.	During HD, samples from the arterial line before the dialyzer; predialysis site NR.	tPA/PAI-1 mass/activity assays	Predialysis PAI-1 mass exceeded the reference range in 63%. D-dimer correlated with tPA activity and TAT, indicating chronic thrombotic burden with secondary fibrinolysis.	(28)
Kushiya et al., 2003	HD patients (*n* = 84) vs. healthy controls (*n* = 20).	Immediately before HD.	Blood; site NR.	NR	tPAIC increased with lower ABI/API and higher ACI, suggesting an association with severe peripheral arterial disease and vascular calcification.	(138)
Kushiya et al., 2003	Chronic renal failure patients (*n* = 133; HD/CT/CAPD) vs. healthy controls (*n* = 73).	Immediately before HD in HD patients.	Blood; site NR.	NR	tPAIC values were comparable across HD, CT and CAPD groups. Higher values were seen in higher Ox-LDL/M-LDL strata, but statistical significance was not reported.	(185)
Segarra et al., 2001	MHD patients (*n* = 200) vs. matched controls (*n* = 100); CAD vs. non-CAD subgroup; validation cohort (*n* = 60).	Before dialysis; fasting predialysis blood for lipid assays.	Antecubital vein specified for lipid testing; coagulation/fibrinolysis sampling site NR.	ELISA; chromogenic assays	PAI-1 Ag/activity, tPA Ag and tPA/PAI complexes were higher in HD patients, whereas tPA activity was unchanged. PAI-1 activity independently predicted CAD.	(122)
Costa et al., 2008	HD patients (*n* = 50) vs. controls (*n* = 25); AVF (*n* = 39) vs. CVC (*n* = 11).	Controls: fasting sample; HD patients: before the second weekly dialysis session.	Blood; site NR.	ELISA	HD patients had lower PAI-1, unchanged tPA and higher tPA/PAI-1 ratio vs. controls. CVC patients had higher tPA, D-dimer and inflammatory markers than AVF patients.	(125)
Trimarchi et al., 2008	HD patients (*n* = 36) vs. controls (*n* = 40); 12-month vascular access thrombosis follow-up.	Fasting predialysis sample; follow-up for access thrombosis.	Blood; site NR.	Chromogenic assay; PCR.	PAI-1 activity was markedly higher in HD patients but did not differ between thrombosis and non-thrombosis groups; access thrombosis clustered in PTFE grafts.	(126)
Marney et al., 2009	Nine chronic HD patients in a randomized, double-blind, placebo-controlled crossover study of HOE-140 vs. vehicle.	Predialysis, HD start, 15 and 30 min during HD, end of HD, and 2 h post-HD.	Serial blood sampling; exact site NR.	ELISA; chromogenic assays	PAI-1 antigen rose at the end of HD and 2 h post-HD and was blocked by HOE-140. tPA antigen/activity increased during/post-HD, while PAI-1 activity decreased.	(128)
Sosinska-Zawierucha et al., 2019	Serum from ESRD HD patients (*n* = 22) before vs. after HD, tested on arterial and venous endothelial cells.	Immediately before HD and after a 4-h HD session.	Serum; site NR.	ELISA; RT-qPCR	Post-HD serum increased venous endothelial tPA secretion and tPA/PAI-1 ratio; responses differed between arterial and venous endothelial cells.	(129)
Opatrný et al., 1998	11 CAPD patients vs. 11 healthy volunteers	Plasma: 0 and 2 h; dialysate: 0, 2, and 4 h during one exchange	Citrated plasma and peritoneal dialysate.	Chromogenic activity; ELISA	Lower plasma tPA activity and dwell-time-dependent dialysate PAI-1 increase suggest systemic hypofibrinolysis and local peritoneal fibrinolytic inhibition in CAPD.	(130)
Misian et al., 2022	CAPD serum vs. healthy serum; CAPD effluent vs. medium; baseline vs. 6-month effluent; ± sulodexide in CAEC model.	Serum and effluent after a 12-h overnight CAPD exchange; repeat effluent in 12 patients at 6 months.	Serum site NR; peritoneal effluent from overnight dwell.	Cell model; ELISA	CAPD serum/effluent increased PAI-1, did not change tPA and reduced tPA/PAI-1 ratio, suggesting a coronary endothelial low-fibrinolytic phenotype.	(131)
Misian et al., 2020	Seven male CAPD patients before/after IV iron isomaltoside (IIS) with or without NAC; *in vitro* mesothelial cell model.	4-h Dianeal 1.5% exchanges before and after IIS; mesothelial cells exposed for 24 h.	Peritoneal effluent; mesothelial cell supernatant.	ELISA; RT-qPCR	IIS increased mesothelial PAI-1 and reduced tPA, lowering the tPA/PAI-1 ratio; NAC prevented these low-fibrinolytic changes.	(132)
Arikan et al., 2009	PD patients grouped by baseline plasma PAI-1: ≤41 ng/mL (*n* = 19) vs. > 41 ng/mL (*n* = 27).	Baseline blood sample; mean follow-up 45.4 months.	Blood; site NR.	ELISA	PAI-1 > 41 ng/mL independently predicted cardiovascular mortality and fatal/nonfatal cardiovascular events in prevalent PD patients.	(133)
Lopes Barreto et al., 2015	EPS cases (*n* = 11) vs. long-term PD controls (*n* = 34).	Annual standard peritoneal permeability tests; samples 1–4 years before EPS diagnosis.	Peritoneal effluent, not blood.	Sandwich ELISA	Effluent PAI-1 appearance rate was persistently higher before EPS and showed moderate early diagnostic performance; MMP-2 was less discriminative.	(134)
Hao et al., 2019	Prospective single-center cohort of incident PD patients (*n* = 70).	Baseline PET at month 1, then every 6 months; focus on baseline, 6-month biomarkers and 2-year PSTR.	Overnight peritoneal effluent, not blood.	Sandwich ELISA	Effluent PAI-1 increased within 6 months. Baseline PAI-1 correlated with baseline and 2-year D/P creatinine and independently predicted 2-year PSTR.	(135)
Onur Cura et al., 2020	PD patients with high/suspected PF risk (*n* = 9) vs. low PF risk (*n* = 16).	Single blood sample at enrollment.	Peripheral blood leukocytes.	PCR; Sanger sequencing	SERPINE1 4G/5G and detected variants were not significantly associated with PF risk; plasma or dialysate PAI-1/tPA/tPAIC was not measured.	(136)
Wang et al., 2013	MODS patients undergoing CBP randomized to heparin anticoagulation (*n* = 8) or heparin-free CBP (*n* = 8), with healthy controls (*n* = 10).	CBP at 0, 15, 60, 120 and 480 min.	Arterial limb of CBP circuit; serum/plasma prepared for assays and cell experiments.	ELISA	PAI-1 decreased while D-dimer increased during CBP; heparin was associated with a greater PAI-1 reduction, suggesting reduced fibrinolytic inhibition.	(137)
Lee et al., 2025	Prospective PD cohort (*n* = 132) classified by PET trajectories: HH, HL, LH and LL; FH vs. FL regrouping.	Baseline PET at enrollment and final PET at study end, PD dropout or death.	PET effluent, not blood.	ELISA	Higher PAI-1 AR was associated with persistent/transitioning high transporter status and poor outcomes. Adding PAI-1/MMP2 AR improved SVM AUC from 0.71 to 0.87.	(187)

### 3.5 Anti-Xa

#### 3.5.1 Detection methods for Anti-Xa activity

Anti-Xa activity measurement is an important laboratory method for evaluating the anticoagulant effect of heparin. In current clinical laboratories, Anti-Xa activity is mainly measured using chromogenic substrate assays (139). This assay is based on the inhibitory effect of the heparin–antithrombin complex on factor Xa in the sample. By measuring the extent to which residual factor Xa cleaves a chromogenic substrate, the anticoagulant activity of the drug can be indirectly quantified (139). Compared with APTT, Anti-Xa assays are less affected by certain biological variables and therefore offer advantages when accurate assessment of heparin anticoagulant intensity is required (140). Anti-Xa assays can be used to monitor UFH and may also be useful for assessing LMWH anticoagulation during IVVHF. However, oral direct factor Xa inhibitors, including apixaban, edoxaban, and rivaroxaban, can falsely elevate UFH-calibrated anti-Xa results. Therefore, when anti-Xa assays are applied across different anticoagulant classes, results should be interpreted with attention to the anticoagulant being measured, assay calibration, and potential drug interference (141, 142).

#### 3.5.2 Clinical significance and dialysis-related applications

Anti-Xa monitoring has been explored in dialysis and blood purification mainly to guide anticoagulant dosing, assess drug accumulation or clearance, and maintain filter or circuit patency. Its use is most established for LMWH, because APTT does not reliably reflect LMWH anticoagulant intensity. However, Anti-Xa testing is not recommended as routine monitoring for all hemodialysis patients; it may be more useful in selected patients with extreme body weight, recurrent circuit clotting, high bleeding risk, impaired renal clearance, or uncertain anticoagulant response (7). In acute extracorporeal therapies, Anti-Xa monitoring is mainly related to filter survival and circuit clotting. In IVVHF, Anti-Xa levels >0.2 IU/mL were associated with less filter and circuit coagulation, supporting its role in LMWH monitoring (142). In CRRT, previous recommendations suggested an LMWH Anti-Xa target of 0.25–0.35 IU/mL, although supporting evidence remains limited (143, 144). Trough Anti-Xa monitoring has also been used to assess possible LMWH accumulation in AKI patients receiving CRRT (145). During CVVH with nadroparin, Anti-Xa activity peaked after bolus dosing and then declined, with no detectable Anti-Xa in ultrafiltrate, suggesting no clear LMWH removal or accumulation by CVVH (146). In critically ill COVID-19 patients receiving CRRT or SLED, Anti-Xa-guided or intensified heparin strategies were feasible and may help reduce filter loss, although evidence remains mixed (147, 148). Anti-Xa testing may also detect systemic heparin exposure after catheter lock use, indicating possible bleeding risk in ICU dialysis patients (149). In maintenance HD, Anti-Xa monitoring has been used mainly to optimize LMWH dosing. Standard-dose LMWH did not show significant accumulation over three consecutive HD sessions, but an Anti-Xa level <0.35 IU/mL at 4 h was associated with higher circuit clotting risk (150). Overall, LMWH appears to have safety comparable to UFH in HD, although accumulation remains a concern in patients with renal impairment (151). Several studies support individualized monitoring rather than universal testing. Nadroparin produced safe Anti-Xa profiles in nocturnal HD without weekly accumulation (152), while population modeling showed that lean body mass, dialysis modality, and dialyzer type influence nadroparin exposure (153). Fixed-dose tinzaparin was effective in most stable conventional HD sessions, supporting simple dosing in selected patients (154). A heparin-coated AN69ST dialyzer enabled HDF with minimal Anti-Xa activity and low severe clotting rates (155). Extended dialysis schedules may require different dosing strategies, as dalteparin was effective in quotidian HD but less successful in nocturnal HD (156). Some studies addressed special kidney-disease populations or non-standard anticoagulants. In severe nephrotic syndrome, fixed-dose enoxaparin often failed to reach target Anti-Xa levels, whereas ideal-body-weight adjustment improved target attainment (157). In CKD patients receiving therapeutic dalteparin, including dialysis patients, higher trough Anti-Xa levels were associated with bleeding, supporting trough monitoring when LMWH accumulation is suspected (158). Fondaparinux anti-Xa levels are influenced by dialysis modality: high-flux HD increased fondaparinux clearance and reduced anti-Xa activity compared with low-flux HD, while limited data suggested that PD had little apparent effect (159). In renal failure or dialysis patients with suspected HIT, a small case series suggested that peak anti-Xa-guided fondaparinux dosing may be feasible when standard alternatives are unavailable (160). During CVVHDF, prophylactic fondaparinux produced higher Anti-Xa activity than controls at some time points, but most patients remained below suggested prophylactic ranges, and clinical significance remains uncertain (161). For DOACs, Anti-Xa results should be interpreted differently from heparin or LMWH monitoring. Drug-specific calibrated Anti-Xa assays are required to estimate concentrations of factor Xa inhibitors such as apixaban, rivaroxaban, and edoxaban (162). Uncalibrated heparin Anti-Xa assays cannot quantify DOAC concentrations, although they may help exclude clinically relevant drug levels in emergencies (163). In HD patients receiving apixaban, drug-level monitoring may support individualized dosing and risk–benefit assessment, although anti-Xa levels should not be used alone to predict bleeding risk (164). In PD patients, the APIDP2 trial will evaluate apixaban anti-Xa activity as a pharmacodynamic outcome, but clinical outcome data are not yet available (165). In HD patients on apixaban, Anti-Xa-measured drug levels increased with dose but did not predict bleeding, whereas ROTEM EXTEM clotting time appeared more informative (166). Overall, Anti-Xa monitoring may help individualize anticoagulation in selected dialysis patients, especially when circuit clotting, bleeding risk, renal impairment, unusual dialysis schedules, or non-standard anticoagulants are present. Its interpretation depends on anticoagulant type, dialysis modality, sampling time, assay calibration, and clinical endpoint. Therefore, Anti-Xa results should be combined with clinical bleeding, circuit outcomes, and other coagulation assessments rather than used in isolation. Table 6 summarizes representative studies on Anti-Xa monitoring in dialysis patients.

**Table 6:** Summary of Anti-Xa monitoring studies in dialysis-related settings.

Author	Population/comparison	Sampling time	Sampling site	Assay	Key findings	References
Sagedal et al., 2001	12 chronic HD (warfarin/non-warfarin) vs. 10 predialysis CRF; 84 HD sessions	1, 3, and 4 h after HD start	Arterial line	Chromogenic assay	End-HD Anti-Xa ≥ 0.4 IU/mL reduced severe circuit/dialyzer clotting.	(178)
Xu et al., 2023	110 patients receiving IVVHF	End of 6-h treatment	Arterial end	Chromogenic substrate	Lower Anti-Xa was associated with more severe filter clotting.	(142)
Oudemans et al., 2009	14 ICU AKI patients receiving CVVH with nadroparin	Pre-CVVH; 1 h; 15 min, 6, 12, and 24 h after flow change	Arterial, postfilter, ultrafiltrate	Chromogenic Anti-Xa	Arterial Anti-Xa peaked then declined; postfilter levels were stable; ultrafiltrate was negative.	(146)
Endres et al., 2021	65 COVID-19 patients receiving CRRT/CVVH	6 h after UFH start or dose change; q12h after target	NR	NR	Initial Anti-Xa target was 0.3–0.5 IU/mL; target was raised to 0.5–0.7 IU/mL if clotting occurred.	(147)
Wen et al., 2020	COVID-19 AKI/SLED (*n* = 52) vs. pre-COVID AKI/SLED (*n* = 51)	During SLED or within +/−3 h	NR	NR	Shortened SLED more often had subtherapeutic Anti-Xa/aPTT than prolonged SLED.	(148)
Bovet et al., 2020	Paired catheter-lock study in 30 patients	Pre-lock and 30 min-1 h post-lock	NR	Colorimetric test	Heparin lock increased Anti-Xa by a median of 0.37 IU/mL, which may confound interpretation.	(149)
Tao et al., 2020	90 maintenance HD patients receiving LMWH	0.5 and 4 h; 3 consecutive HD sessions	Afferent line	STA Compact Max	Anti-Xa peaked at 0.5 h, declined by 4 h, and showed no clear accumulation.	(150)
Buitenwerf et al., 2015	13 nocturnal in-center HD patients	First HD pre; last HD pre/post; next-week pre	NR	Photometric chromogenic	Anti-Xa increased after the last HD session but returned to baseline the next week.	(152)
Jaspers et al., 2022	137 dialysis patients receiving nadroparin	0/5/30/60/180/240 min	NR	Chromogenic assay	Only 38.4% of patients had end-HD Anti-Xa ≥0.4 IU/mL.	(153)
Kirwan et al., 2013	43 maintenance HD patients receiving tinzaparin	0, 30, 60, 120 min, and end-HD	NR	Chromogenic FXa inhibition	Baseline Anti-Xa was undetectable; all end-HD levels were <0.4 IU/mL.	(154)
Sánchez-Canel et al., 2012	6 maintenance dialysis patients; AN69ST without systemic anticoagulation vs. standard dialyzer + nadroparin	Start, 2 h, and end-HD	Arterial branch	NR	AN69ST maintained low systemic Anti-Xa; standard anticoagulation increased Anti-Xa.	(155)
Huang et al., 2020	18 HD patients switched to dalteparin	Day 7 after start/dose change; follow-up to 12 months	NR	Chromogenic Anti-Xa	Trough Anti-Xa remained <0.1 IU/mL in continuing patients; no major bleeding occurred.	(156)
Matyjek et al., 2021	Severe nephrotic syndrome: fixed vs. weight-based enoxaparin; non-proteinuric controls	4 h post-dose	NR	Chromogenic assay	Fixed dosing often underachieved target Anti-Xa; weight-based dosing improved target attainment.	(157)
Gurumurthy et al., 2026	eGFR <30 receiving therapeutic dalteparin; 78 monitored	Trough <=1 h pre-dose; peak 3–5 h post-dose	Venous blood	Chromogenic assay	Higher trough Anti-Xa was independently associated with bleeding; ≥ 0.25 U/mL indicated higher risk.	(158)
Michaličková et al., 2022	12 dialysis-dependent CKD patients (11 on HD) receiving fondaparinux	Based on admission, treatment timing, and steady state	NR	Chromogenic assay	High-flux HD increased fondaparinux clearance 2.26-fold; low-flux HD and PD had limited effect.	(159)
Ghaziri et al., 2023	10 renal failure patients with suspected HIT requiring anticoagulation	4 h post-fondaparinux; less often once stable	NR	NR	Dose adjustment by 4-h Anti-Xa achieved target levels in all patients without supratherapeutic values.	(160)
Harvey et al., 2023	47 nocturnal home HD training patients	0, 15, 30 min; 1, 2, 4, 6, and 8 h	Arterial circuit line	Colorimetric assay	Anti-Xa peaked at 15 min and declined gradually; low activity remained detectable at 8 h.	(188)
Aszkiełowicz et al., 2025	CVVHDF (*n* = 20) vs. non-CVVHDF (*n* = 20) receiving fondaparinux	Day 3: 0, 3, 6, and 9 h	Arterial blood	STA-Liquid Anti-Xa	Most levels were below the prophylactic range; CVVHDF levels were slightly higher at some time points.	(161)
Ficheux et al., 2024	PD with nonvalvular AF: apixaban vs. warfarin	M1, M6, M12; bleeding events	NR	NR	Apixaban Anti-Xa monitoring was recorded; detailed outcome data were not available in the source table.	(165)
Bacharaki et al., 2025	19 chronic HD patients with nonvalvular AF using apixaban ≥ 8 days	Pre-dose trough and 2 h post-dose peak	Peripheral venous blood	Chromogenic assay	Apixaban levels were measurable, but Anti-Xa drug concentration did not predict bleeding.	(166)
Batt et al., 2020	16 chronic ESRD HD patients receiving enoxaparin	0, 2, 4 h, and end-HD	NR	Colorimetric assay	Anti-Xa peaked at 2 h; therapeutic Anti-Xa rates declined by 4 h and end-HD.	(189)

## 4 Value of multimarker combined monitoring in clinical decision-making

Multimarker or ratio-based approaches have been used to assess coagulation-fibrinolysis imbalance more comprehensively than single markers. In hemodialysis patients, Hasuike et al. reported that an elevated TAT/PIC ratio was independently associated with vascular access failure after vascular access intervention, suggesting that coagulation–fibrinolysis imbalance may be relevant to dialysis-related vascular access outcomes (84).

Outside dialysis, combined-marker strategies have also been evaluated in thrombotic or coagulopathic conditions. Mei et al. showed that TAT, PIC, tPAIC, and sTM had diagnostic and prognostic value in DIC (167). Zhou et al. found that the combination of TAT, PIC, TM, tPAIC, D-dimer, and FDP improved VTE diagnosis in malignant tumor patients compared with single markers (63). Cheng et al. further reported that the postoperative day-6 TAT/PIC ratio showed high sensitivity and negative predictive value for early VTE detection after joint arthroplasty, although its low specificity means that imaging confirmation remains necessary (168). However, dialysis-specific evidence remains limited. Current data mainly support the association between the TAT/PIC ratio and vascular access failure, whereas validated four-marker algorithms for anticoagulant adjustment, circuit clotting prediction, bleeding-risk assessment, or access-intervention decisions have not yet been established in dialysis patients (84). Thus, multimarker monitoring should currently be viewed as an adjunctive and exploratory strategy rather than an established decision-making tool. Although automated chemiluminescence platforms allow reliable measurement of TM/sTM, TAT, PIC/PAP, and tPAIC (57, 92).

## 5 Challenges in the clinical application of multimarker monitoring: pre-analytical variables and platform standardization

Although these biomarkers have distinct mechanistic advantages, their interpretation may be affected by pre-analytical handling and assay/platform differences, including venous-stasis effects on tPA/tPAIC, reagent variability in anti-Xa assays, and inter-kit differences in sTM/TM measurement (169–172). Pre-analytical factors, including collection tube type, anticoagulant selection, centrifugation conditions, transportation procedures, and storage temperature, may introduce substantial analytical error and result variability (173). Chen et al. showed that TM, TAT, PAP, and tPAIC assays on the HISCL-5000 had acceptable analytical performance, but prompt post-centrifugation testing, avoidance of frozen storage, and local verification of tPAIC reference intervals remain necessary (92). Tang et al. further evaluated the performance of the Sysmex HISCL-5000 high-sensitivity chemiluminescence analyzer for measuring TM, TAT, PIC, and tPAIC. Their findings showed that both within-run and between-day coefficients of variation were below 5%, with good linear correlations for all measured parameters, supporting their analytical suitability for clinical specimens (57). Nevertheless, methodological differences remain across platforms. Gardiner et al. showed that CLEIA assays for TM, TAT, tPAIC, and PIC on the Sysmex CN-6500 had good analytical performance and correlated well with the HISCL-800, but tPAIC bias and local reference interval requirements still support method comparison and local validation (174). In addition, Zhang et al. established reference intervals for TAT, PIC, TM, and tPAIC in 1,628 healthy older Chinese adults, showing that TAT varied by age and sex, while PIC was mainly age-related. This supports population-specific interpretation rather than fixed universal thresholds (76).

The analytical challenges of Anti-Xa activity are somewhat different. Chiasakul et al. noted that UFH can be monitored using APTT, ACT, or Anti-Xa activity, whereas LMWH monitoring mainly relies on Anti-Xa activity. However, routine laboratory monitoring has not been shown to improve clinical outcomes in most hemodialysis patients and is therefore generally not recommended for universal use. Instead, it is more appropriate for selected patients, such as those with extreme body weight, recurrent extracorporeal circuit clotting, or high bleeding risk (7). For Anti-Xa testing, standardization mainly involves collection tube type, sampling time, reagent platform, and calibration system. A study evaluating the stability of Anti-Xa activity in citrate and citrate-theophylline-adenosine-dipyridamole (CTAD) tubes reported that Anti-Xa activity has become an important reference assay for heparin monitoring and found that samples were relatively stable within 6 h under certain conditions. However, differences in reagents, collection tubes, and analytical workflows may still affect result interpretation (175). For direct factor Xa inhibitors, the 2021 International Council for Standardization in Haematology (ICSH) update emphasized that appropriate laboratory assays and calibration systems should be selected according to the specific drug being assessed (176). Therefore, the central challenge of multimarker monitoring is not merely determining which biomarkers should be measured, but ensuring that different biomarkers are comparable and interpretable within the same clinical context.

## 6 Practical framework for biomarker-guided monitoring in dialysis-related thrombosis and anticoagulation

The clinical use of molecular biomarkers in dialysis patients should be scenario-based rather than universal. Current evidence does not support routine measurement of all biomarkers in all maintenance hemodialysis patients. For heparin-related monitoring, laboratory testing may be considered in selected patients with extreme body weight, recurrent extracorporeal circuit clotting, high bleeding risk, suspected under- or over-anticoagulation, or concern for LMWH accumulation (7, 56, 150). Other scenarios, such as shortened CRRT filter life, recurrent vascular access dysfunction, and acute inflammatory or septic states, may justify targeted biomarker assessment, but require further dialysis-specific validation.

Anti-Xa activity is directly actionable for heparin-related pharmacodynamic monitoring. In HD patients receiving LMWH, a 4-h anti-Xa level <0.35 IU/mL was associated with increased ECC clotting, while in IVVHF, anti-Xa > 0.2 IU/mL was associated with lower filter and line coagulation grades (142, 143, 150).

In patients receiving CRRT, recurrent filter clotting should first prompt a systematic assessment of non-pharmacological factors, including vascular access function, catheter position, blood flow rate, pre−/post-dilution strategy, hemoconcentration, membrane characteristics, and circuit configuration (6, 36). When no contraindication to citrate exists and appropriate monitoring is available, regional citrate anticoagulation should be considered preferentially over systemic heparin anticoagulation for CRRT, as suggested by KDIGO and supported by randomized clinical evidence showing longer filter lifespan with citrate than with systemic heparin (4, 31, 143).

In dialysis patients, TAT and PIC/PAP should be interpreted primarily as pathophysiological markers of coagulation–fibrinolysis balance rather than direct triggers for anticoagulant dose escalation. Hasuike et al. showed that TAT reflects thrombin generation, PIC reflects plasmin generation, and an elevated TAT/PIC ratio was independently associated with vascular access failure after intervention (84). sTM should be interpreted mainly as a surrogate marker of endothelial injury or endothelial dysfunction, rather than as a thrombosis-specific or event-specific marker (13, 110, 111). The tPAIC may indicate PAI-1-mediated tPA inactivation and impaired fibrinolytic regulation, but should be interpreted together with PIC/PAP, D-dimer, PAI-1, inflammatory markers, and clinical findings (76, 177).

Sampling timing and blood source should be standardized before these biomarkers are used for clinical interpretation. Baseline thrombotic risk assessment should preferably use samples collected before dialysis and before heparin administration. Dynamic assessment during hemodialysis may include repeated sampling during or near the end of the session to evaluate dialysis-induced coagulation and fibrinolytic activation. For LMWH monitoring, Anti-Xa should be measured at a predefined post-dose time point, and trough sampling may be considered when drug accumulation is suspected. In CRRT, the sampling site should be explicitly defined as systemic, pre-filter, or post-filter blood because these sources reflect different biological and pharmacological processes. Sampling from heparin-containing catheter locks or infusion lines should be avoided whenever possible because heparin contamination may lead to misleading coagulation or Anti-Xa results (92, 176).

## 7 Conclusion

Dialysis patients have a complex hemostatic profile characterized by simultaneous thrombotic risk, bleeding tendency, endothelial injury, inflammation, extracorporeal circuit activation, and anticoagulant exposure. Conventional tests such as PT, APTT, ACT, and D-dimer provide limited information and cannot fully reflect the dynamic coagulation–fibrinolysis imbalance in this population. TAT, PIC/PAP, sTM, tPAIC, and Anti-Xa activity provide mechanistically complementary information. TAT reflects thrombin generation, PIC/PAP indicates fibrinolytic activation, sTM reflects endothelial injury, tPAIC suggests impaired fibrinolytic regulation, and Anti-Xa activity evaluates anticoagulant drug effect. However, these biomarkers should currently be interpreted as adjunctive and exploratory tools rather than as established decision-making markers for dialysis-related thrombosis or anticoagulation management. At present, there is no validated five-marker algorithm in dialysis patients, and outcome-based evidence remains limited for several individual biomarkers.

However, routine clinical application remains limited by pre-analytical variability, assay standardization, uncertain reference intervals, cost-effectiveness concerns, and insufficient prospective outcome data. Future multicenter studies are needed to validate standardized multimarker strategies and clarify their role in vascular access protection, circuit patency, bleeding prevention, and individualized anticoagulation management.

## Glossary

ABI: Ankle-brachial index
ACI: Aortic calcification index
ACT: Activated clotting time
ADAMTS13: A disintegrin and metalloproteinase with thrombospondin type 1 motif, member 13
AF: Atrial fibrillation
AHR: Aryl hydrocarbon receptor
AKI: Acute kidney injury
AN69ST: Surface-treated AN69 membrane
Anti-Xa: Anti-factor Xa activity
APTT: Activated partial thromboplastin time
ARDS: Acute respiratory distress syndrome
AUC: Area under the curve
AVF: Arteriovenous fistula
AVG: Arteriovenous graft
CAEC: Coronary artery endothelial cell
CAPD: Continuous ambulatory peritoneal dialysis
CBP: Continuous blood purification
CD11b: Cluster of differentiation 11b
CD62P: P-selectin
CKD: Chronic kidney disease
CLEIA: Chemiluminescent enzyme immunoassay
CLIA: Chemiluminescence immunoassay
CRF: Chronic renal failure
CRP: C-reactive protein
CRRT: Continuous renal replacement therapy
CTAD: Citrate-theophylline-adenosine-dipyridamole
CVC: Central venous catheter
CVVH: Continuous venovenous hemofiltration
CVVHDF: Continuous venovenous hemodiafiltration
DIC: Disseminated intravascular coagulation
DOAC: Direct oral anticoagulant
eGFR: Estimated glomerular filtration rate
EIA: Enzyme immunoassay
ELISA: Enzyme-linked immunosorbent assay
EPO: Erythropoietin
EPS: Encapsulating peritoneal sclerosis
ESRD: End-stage renal disease
EXTEM: Extrinsically activated thromboelastometry assay
F1 + 2: Prothrombin fragment 1 + 2
FDP/FDPs: Fibrin/fibrinogen degradation products
FIB: Fibrinogen
FVII: Coagulation factor VII
FVIII: Coagulation factor VIII
FXa: Activated factor X
GDPs: Glucose degradation products
HD: Hemodialysis
HDF: Hemodiafiltration
HIT: Heparin-induced thrombocytopenia
HUVEC: Human umbilical vein endothelial cell
IC50: Half-maximal inhibitory concentration
ICSH: International Council for Standardization in Haematology
ICU: Intensive care unit
IIS: Iron isomaltoside
INR: International normalized ratio
IVVHF: Intermittent venovenous hemofiltration
KDIGO: Kidney Disease: Improving Global Outcomes
KFRT: Kidney failure receiving replacement therapy
LMWH: Low-molecular-weight heparin
MAPK: Mitogen-activated protein kinase
MHD: Maintenance hemodialysis
M-LDL: Malondialdehyde-modified low-density lipoprotein
MMP-2: Matrix metalloproteinase-2
MODS: Multiple organ dysfunction syndrome
NAC: N-acetylcysteine
NF-κB: Nuclear factor kappa B
OR: Odds ratio
Ox-LDL: Oxidized low-density lipoprotein
PAF: Platelet-activating factor
PAI-1: Plasminogen activator inhibitor-1
PAP: Plasmin–antiplasmin complex
PD: Peritoneal dialysis
PET: Peritoneal equilibration test
PF: Peritoneal fibrosis
PIC: Plasmin–α2-antiplasmin complex
PMMA: Polymethylmethacrylate
PP: Polypropylene
PT: Prothrombin time
PTA: Percutaneous transluminal angioplasty
PTFE: Polytetrafluoroethylene
PSTR: Peritoneal small-solute transfer rate
ROTEM: Rotational thromboelastometry
RT-PCR: Reverse transcription polymerase chain reaction
RT-qPCR: Reverse transcription quantitative polymerase chain reaction
SBP: Systolic blood pressure
SERPINE1: Serpin family E member 1
SIC: Sepsis-induced coagulopathy
SLED: Sustained low-efficiency dialysis
sTM: Soluble thrombomodulin
SVM: Support vector machine
T50: Serum calcification propensity half-time
TAT: Thrombin–antithrombin complex
TEG: Thromboelastography
TF: Tissue factor
TFPI: Tissue factor pathway inhibitor
TGA: Thrombin generation assay
TM: Thrombomodulin
tPA: Tissue-type plasminogen activator
tPAIC: Tissue-type plasminogen activator–plasminogen activator inhibitor-1 complex
u-PA: Urokinase-type plasminogen activator
UFH: Unfractionated heparin
VTE: Venous thromboembolism
vWF: von Willebrand factor
vWF:Ag: von Willebrand factor antigen
vWF:RCo: von Willebrand factor ristocetin cofactor activity
